# Supplementary material for: Learning curve and surgical outcome of robotic assisted colorectal surgery with ERAS program
Source: Sci Rep. 2022 Nov 29;12:20566. doi: 10.1038/s41598-022-24665-w (PMC9709162; doi:10.1038/s41598-022-24665-w)
Supplement: Supplementary file 2 — Supplementary Information 2. [file 41598_2022_24665_MOESM2_ESM.zip › RAL Raw data-Ñ[▒K/121/HS_ñw╝╨░O▒Kñσ.pdf]

正式摘要(電子簽章：000000228696879)

W52 / W52 ☐ 英文標題(注意:無英文姓名-請補病人基本檔!) ☐【讀取】憑證

臺中榮民總醫院

出院病歷摘要

轉入號： 轉出號：

[redacted]

**(8)**流水編號	
**(9)**入院日期	110/10/27 (2021)
**(10)**轉科日期	
**(11)**出院日期	110/11/01 (2021) 住院天數計 5 日

診斷

- 入院：1. S-colon tumor, cT4NxM1c
  2. Hyperlipidemia
- 出院：1. S-colon tumor, cT4NxM1c s/p Robotic assisted LAR on 2021/10/28
  2. Newly dignosed DM, HbA1C:7.9
  3. Hyperlipidemia

主訴

- constipation habit change and estimated for surgery

病史

- This is a 66 year- old men with underline disease of

  # pre-DM

  # Keloid

  This time he was admitted to our ward for constipation habit change and estimated for surgery.

  Tracing back to his history, his was suffered from bowel habit change(small caliber stool) and ocassionally mild bloody stool in recent 6 months. He denied abdominal pain, body weight loss,tenesmus ,rectal mass sensation,dizness, dyspnea.

  Owing to frequent and liquid stool passage, he came to our ER on 10/14. 10/14 colonscopy showed Colon cancer, S-colon, with nearly total obstruciton and oozing. Pathology repost showed invasive adenocarinoma. Abdominal CT revealed focal asymmetrical wall thickening at S-colon.Mild elevated CEA (5.68) and normal CA199 were also noted.

  Under suspicion of Colon cancer,he was admitted to our ward and estimated to do Robotic assited colectomy tomorrow.

體檢發現

-
  - General appearance :
    - easy looking , conscious clear , without cardiopulmonary distress
    - body weight : 59.5 kg , height : 165.6 cm
    - Vital signs自動帶入date 體溫(BT) 脈搏(P) 呼吸(R) 血壓(BP) 身高(BH) 體重(BW) BMI
      20211027 36℃ 86次/分 18次/分 160/76mmHg 165.6cm 59.9kg 21.8
    - Vital signs: B.P. 160/7 mmHg , P.R. 86 /min , R.R. 18 /min , B.T. 36 ℃
  - HEENT :
    - Eyes: no anemic conjunctiva, no icteric sclera, normal EOM
    - ENT: no otorrhea, no rhinorrhea, no erythematous mucosa, no injected tonsil, no deformity
  - Neck: supple, freely movable, trachea midline,no deformity, no engorged jugular vein , no palpable thyroid or lymph node enlargement
  - Chest:
    - Inspection: Grossly normal, symmetric and fully expansion, no accessory muscle used, no operation scar, no spider angiomata, no gynecomastia
    - Palpation: no palpable mass or nodule, no subcutaneous emphysema, no tenderness
    - Ausculation: clear breathing sound
  - Heart:
    - Palpation: no thrill, no heave
    - Percussion: normal heart size
    - Ausculation: regular heart beat, normal S1, S2, no gallop, no murmur

-
  - Extremities: No deformity, full ranged of motion without limitation, no clubbing finger or nail cyanosis, no pitting edema, no ulcer or pigmentation, no obvious skin lesion, no varicose vein, no bone, joint or muscle tenderness

CRS CONDITION

- Karnofsky performance status: (100) Normal, no complaints, no evidence of disease
- PE of Abdomen:
- __1. Inspection: flat
- __2. Ausculation: normoactive
- __3. Palapation: tympanic
- __4. Percussion: soft, no tenderneness
- CEA: 5.68 ng/ml
- Imaging Studies:
- Colonoscope report: Colon cancer, S-colon, with nearly total obstruciton and oozing
- Sono , CT of abdomen and chest Films:focal asymmetrical wall thickening at S-colon
- Figures:
  1. colon & rectum

手術日期及方法

- 手術日期及方法20211028 Robot Assisted Colectomy(L)
- 手術結果及發現：

住院治療經過

- After admission, Robotic assisted LAR was done on 2021/10/28. Under ERAS protocol, he start try bland I diet since 10/29, and remove Foley tube then start try Bland II diet since 10/30. Due to well appetite without discomfort, we let him discharge on 2021/11/01 and arrange OPD follow up on 2021/11/09.

合併症

一般檢查

- 累積報告：

DATE	TIME	ALB	TP	BIL.T	BIL.D	ALKP	AST	ALT	LDH	AMY	LIPASE	NH3	Ferritin	UA
1101014	1718	3.9		1.1			28	29						
1101019	1114	4.1		0.51	0.14	141	37	45	179					
1101027	1552	3.9	7.0	0.45	0.10		34	35						2.5
1101028	1944	3.6		0.75	0.20		28	33						

DATE	TIME	WBC	RBC	HGB	HCT	MCV	PLT	BLST	NEUT	BAND	SEG	LYM	MON	EOS	BAS
1101014	1718	5550		11.6	33.1	83.2	197				62.0	27.0	3.0	8.0	0.0
1101019	1114	6080	4.14	12.0	34.1	82.4	246				45.0	28.0	9.0	18.0	0.0
1101027	1548	5000	3.98	11.6	33.5	84.2	194		53.0			26.2	6.8	13.4	0.6
1101028	1943	7720		11.1	31.2		171		89.0			8.9	1.0	1.0	0.1

DATE	TIME	NA	K	CL	BUN	CREAT	eGFR	CA	Mg	P	CRP	ESR	PCT	D-Dimer	LACTATE
1101014	1718	122	4.0	89	9	0.81	101.33	8.5			0.430				8.3
1101019	1114	121	4.6		15	1.23		9.1							
1101027	1552	125	4.2		18	0.79	104.3	8.6							
1101028	0606	131													
1101028	1944	126	3.5		11	0.72	116.09	7.9							10.4

DATE	TIME	GLUCOSE	HbA1C	CHOL	TG	HDL	LDL	KETONE	CK	CKMB	TRO-T	PT-P	APTT-P	NT-pro BNP
1101014	1718	138										11.3	33.3	
1101019	1114	133										10.9	32.0	
1101027	1551		7.9											
1101027	1552			214	232	44	143							

DATE	TIME	SP-GR	PH	PROT	GLUC	KETON	BILI	UROB	OB	NITR	LEU	RBC	WBC	EPCEL
1101014	1809	1.013	7.0	Negative	Negative	1+	Negative	0.2	Negative	Negative	Negative	0-2	0-2	0-1

DATE	TIME	PH	BEB	SCVO2	PO2	HCO3	HGB	PCO2	TCO2
1101014	1718	7.364	-0.9	51.3	32.7	24.6		44.2	25.9
1101028	1944	7.358	-4.2	99.6	142.1	20.8		37.8	21.9

特殊檢查

病理報告

其它

出院時情況

- 3.改門診治療

出院指示

藥名	劑量	劑量單位	用法	頻次	數量	數量單位	現況	天數	單位
Xanax tab 0.5mg	1	TAB	PO	QN	8	TAB	TH	8	天
500mg Glucophage	1	TAB	PO	BID	16	TAB	TH	8	天
(□)Takepron OD tab 30mg	1	TAB	PO	QDAC	8	TAB	TH	8	天
Acetylcysteine tab	1	TAB	PO	QN	8	TAB	TH	8	天
XyzaL tab 5mg	1	TAB	PO	QD	8	TAB	TH	8	天

出院計劃

- 門診日期
  - date on 2021/11/09
- Arrange OPD follow up Pending pathology report

主治醫師 2062F 蔣鋒帆 / 住院醫師 2339C 蔡濬樟

正式摘要(電子簽章：000000228696879)

W52 / W52

doctorRank:B
